# Extraosseous osteochondroma of superficial fascia layer of the heel: A case report and review of literature

**DOI:** 10.1097/MD.0000000000032014

**Published:** 2022-12-09

**Authors:** Shaobo Zhu, Junhao Zeng, Zhi Zhang, Cunmin Rong

**Affiliations:** a School of Clinical Medicine of Jining Medical University, Jining, China; b Department of Hand & Foot Surgery, Affiliated Hospital of Jining Medical University, Jining, China.

**Keywords:** diagnosis and treatment, extraskeletal osteochondroma, soft tissue tumor

## Abstract

Osteochondroma is a common benign bone tumor consisting of cartilage-covered bone confluent with the medullary canal of the epiphysis. Extraosseous osteochondroma shares the same appearance and histologic features as a typical osteochondroma but does not have any attachment to surrounding bone structures. Because of its low incidence, extraosseous osteochondroma is uncommon in clinical workups and thus prone to misdiagnosis. The diagnosis of extraosseous osteochondroma should be considered when there is a well-defined bony mass in the soft tissue with no direct continuity with the adjacent bone or joint. Here, we present a case of an imaging diagnosis of “calcified bursitis in the subcutaneous superficial fascial layer” and a postoperative pathological diagnosis of “extraosseous osteochondroma.”

**Patient concerns:**

The patient was a 61-year-old man who had a right plantar heel mass for 2 years and recently visited the hospital because of discomfort in shoes.

**Diagnoses:**

The patient was diagnosed with pathological examination.

**Interventions:**

After completing the relevant preoperative examination and preoperative preparation and excluding contraindications to surgery, surgery was performed under nerve block anesthesia.

**Outcomes:**

We performed surgical resection, and the patient did not have obvious discomfort when discharged from the hospital. Auxiliary examination showed no abnormalities.

**Lessons:**

For foot tumors, we need to consider the possibility of extraosseous osteochondroma. After completing the auxiliary examination, we should determine the relationship between the tumor and its surrounding tissues and blood supply before surgery to avoid causing major trauma.

## 1. Introduction

Osteochondroma is common and usually found in the epiphysis of long bones with a cartilage cap and continuation with the medullary cavity of long bones. Soft tissue osteochondroma is uncommon, which may be found in the synovial tissue of joints, tendon sheaths, or bursae. Only a few cases of extraosseous osteochondroma of the foot have been reported. Here, we report a case of extraosseous osteochondroma in the superficial fascial layer of the heel.

## 2. Case report

**Figure 1.**
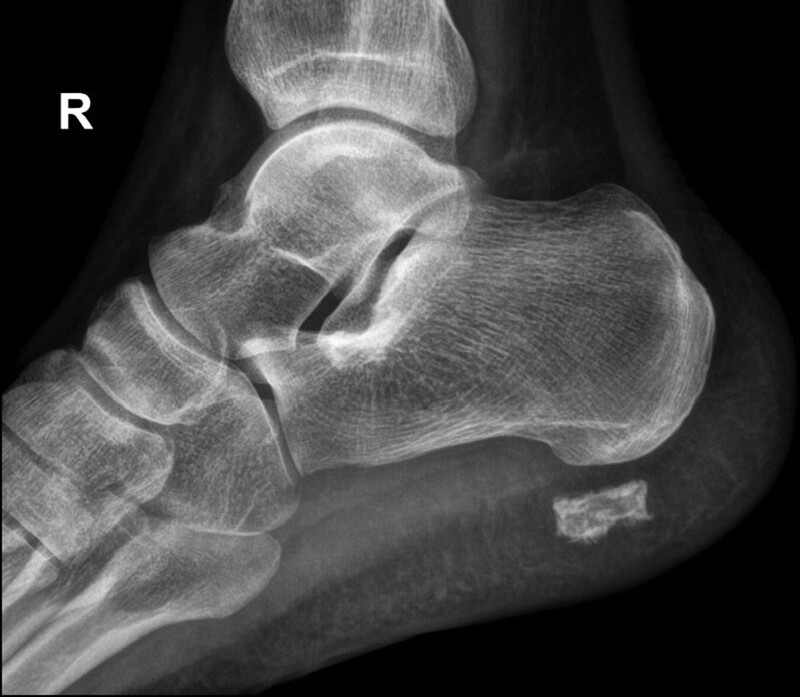
Right heel X-ray showing a patchy high-density shadow in the soft tissue of the lower edge of the right heel.

**Figure 2.**
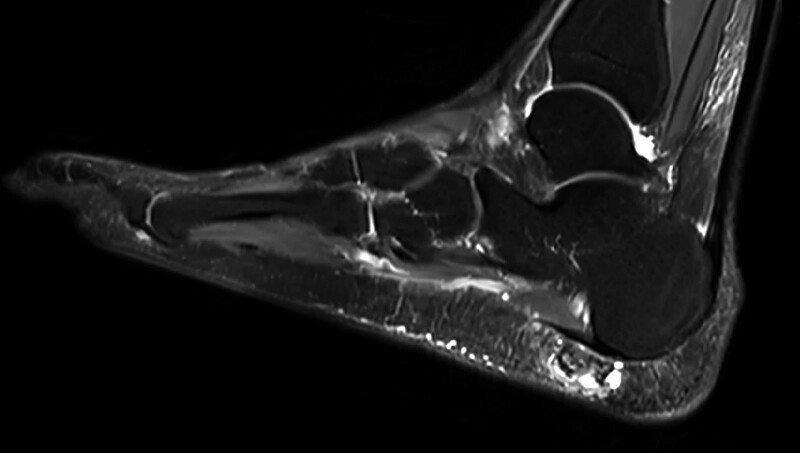
MRI scan of the right foot showing an abnormal signal shadow in the superficial fascial soft tissue of the right sole. MRI = magnetic resonance imaging.

The patient was a 61-year-old man who had a right plantar heel mass for 2 years and recently came to the hospital because of discomfort in shoes. On examination, the skin of the right heel plantar was not red, swollen, or broken. The skin was locally elevated, and a sub-circular swelling of around 2 cm in diameter could be palpated with unclear borders, tough texture, painful pressure, no obvious blood flow pulsation, and poor mobility. The foot and ankle were mobile with good peripheral sensation and blood flow. The patient underwent ultrasound examination at an external hospital, which showed that the plantar bursa was enlarged in the subcutaneous superficial fascial layer of the right plantar foot, and a strong echogenic mass was detected in the bursa with poorly defined borders, an irregular shape, and acoustic shadowing. The continuity of the Achilles tendon and plantar fascia of the right foot was intact, and no thickening was observed. Ultrasound suggested calcified bursitis in the subcutaneous superficial fascial layer of the right plantar foot. After admission to our hospital for surgical treatment, right heel X-ray was performed, which showed a patchy high-density shadow in the soft tissue of the lower edge of the right heel (Fig. 1). Magnetic resonance imaging (MRI) of the right foot showed an abnormal signal shadow in the superficial fascial soft tissue of the right sole with a mixed high/low signal intensity on T1-weighted images (T1WI) and T2-weighted images (T2WI), a predominantly low signal intensity, and a ring-shaped low signal shadow at the edge (around 18 × 12 × 17 mm in size). There were clear margins and a small amount of fluid signal at the posterior edge with slightly swollen adjacent soft tissue. The remaining bones of the right foot showed no obvious abnormal signal, and the surrounding soft tissues showed no obvious abnormalities (Fig. 2).

**Figure 3.**
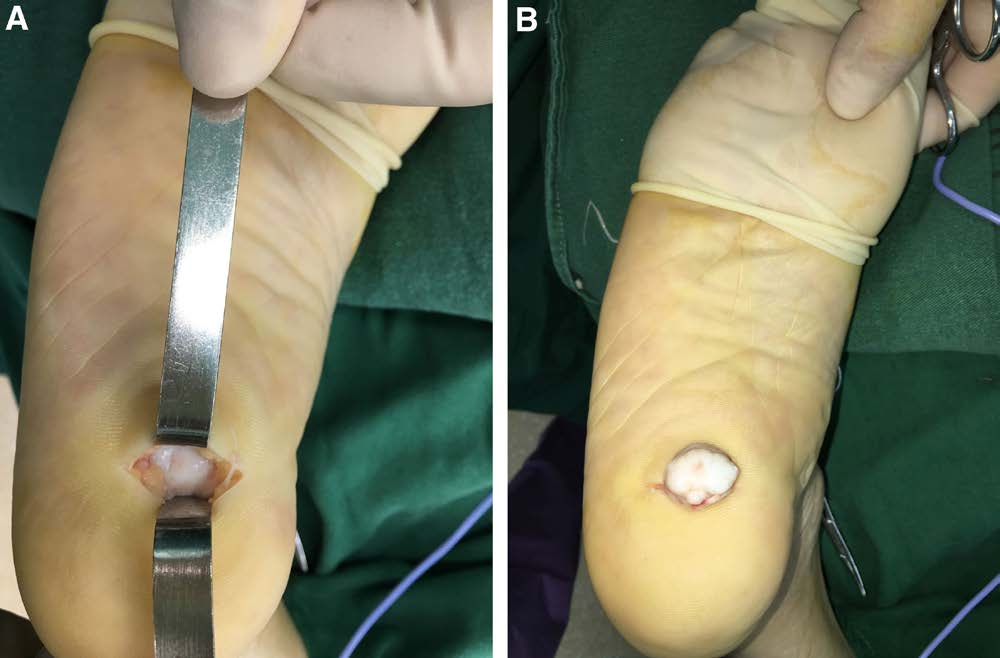
Appearance of the tumor after skin incision during operation.

**Figure 4.**
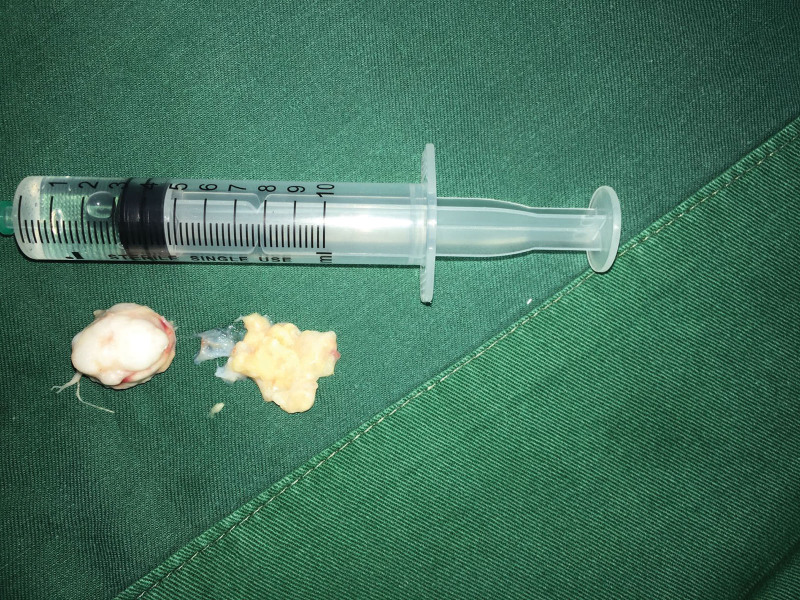
Flexible tumor with an incomplete capsule after complete resection.

**Figure 5.**
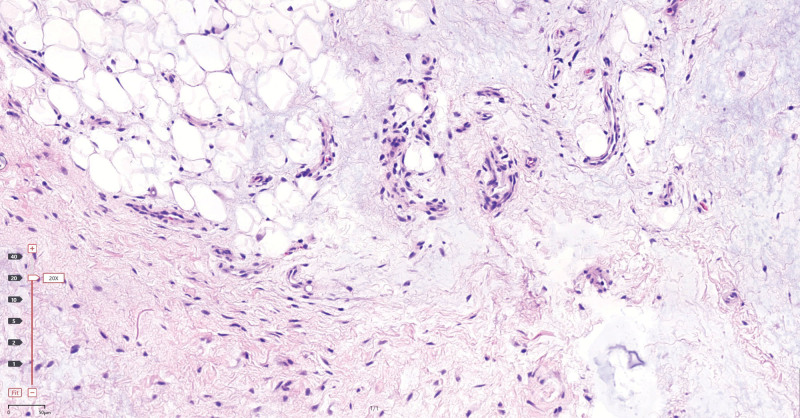
Histopathologic examination of the mass showing mature chondroid tissue (hematoxylin & eosin, 20×).

**Figure 6.**
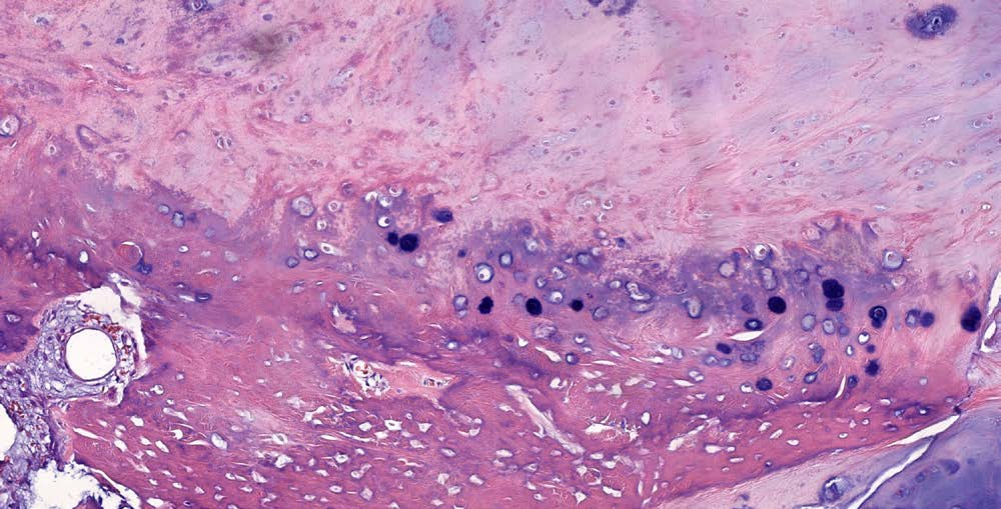
Microscopic examination of the same mass showing the foci of ossified tissue (hematoxylin & eosin, 200×).

**Figure 7.**
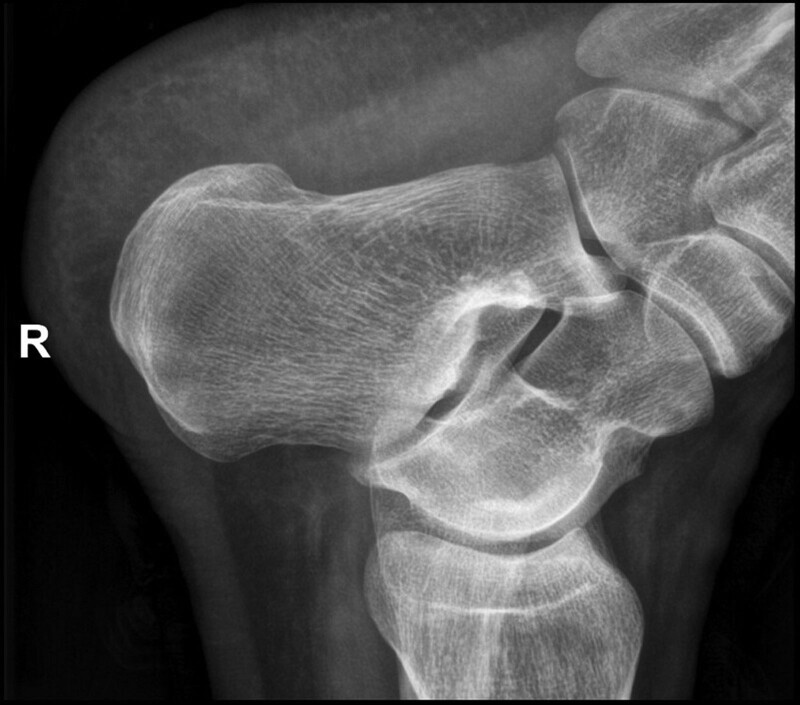
Right heel X-ray showing the intact bone structure of the right heel without obvious bone abnormalities.

After completing the relevant preoperative examination and preoperative preparation and excluding contraindications to surgery, surgery was performed under nerve block anesthesia, and a transverse incision of around 4 cm was made on the surface of the right plantar mass along the skin line (Fig. 3), cut the skin and subcutaneous tissue, and saw the hard tumor in the fat pad of the heel (around 2 × 2 cm in size, the surrounding synovium is hyperplastic and the capsule is incomplete), completely separate and remove the tumor (Fig. 4). Postoperative pathological examination (right plantar) was consistent with osteochondroma and hyperplastic synovial tissue (Figs. 5 and 6). Postoperative X-ray examination showed that the bone structure of the right heel was intact, no obvious bone abnormalities were observed, and the indicated joint space was available (Fig. 7). The patient was discharged 6 days after surgery with good recovery of blood flow function of the affected limb and no significant discomfort.

## 3. Discussion

Extraosseous osteochondroma, also known as soft tissue osteochondroma, is a relatively rare, slow-growing benign soft tissue tumor that is usually found in the soft tissues of the hands and feet and presented as a small, discrete calcified mass rarely exceeding 2 to 3 cm in size.^[1,2]^ Therefore, clinical manifestations are not obvious; however, painful swelling and discomfort become more pronounced as the mass gradually increases in size.

On imaging, the X-ray presentation of an extraosseous osteochondroma is a well-defined lobulated soft tissue mass with a central calcified or ossified area,^[3]^ and the mass is not attached to the periosteum or bone cortex, nor is it within the confines of the joint capsule or tendon sheath. Computed tomography will likewise show the specific location and central calcification or ossification of extraosseous chondroma.^[4,5]^ MRI is the best method to determine the boundaries of the mass, which shows a well-defined heterogeneous lesion with a mostly low signal intensity on T1WI and a mixed high and low signal intensity on T2WI. The T2 signal is moderately intense in areas of mature ossification and low in areas of high-density calcification.^[3–6]^ In addition, MRI can be used to determine the size of the unsettled cartilage cap; the cartilage cap shows a low-to-moderate signal intensity on T1WI and a high signal intensity on T2WI.^[3–6]^

The exact mechanism of the disease is unclear. Nevertheless, it is thought that the mesenchymal cells or fibroblasts in the interstitial matrix of the muscle tissue are stimulated by intrinsic or extrinsic factors, and they are transformed into chondroblasts or osteoblasts, gradually forming osteochondroma. Another hypothesis is the displacement of epiphyseal chondrocytes, resulting in new endochondral bone growth in the soft tissue. Some reports have suggested that it may be related to the chemotactic transformation of lipoma or endochondral osteogenesis.^[3,6–8]^

In this case, the patient visited the hospital with discomforting heel plantar swelling and was diagnosed with calcific bursitis on imaging during the first ultrasound examination. Extraosseous osteochondroma needs to be differentiated from synovial osteochondromatosis, myositis ossificans, periosteal chondroma, and tumoral calcific deposits. Synovial chondromatosis is usually characterized by multiple cartilage and osteochondral nodules within the synovial membrane and can also manifest as intra-articular free bodies. Synovial chondromatosis is identified based on the presence of subsynovial cartilage growth.^[2,9]^ Myositis ossificans is usually heterogeneous or amorphous in its early stages and can increase or decrease in size within a few weeks, typically presenting as a “banding phenomenon” of peripheral calcification consistent with muscle alignment and without the presence of a clear cartilage zone around the osteoclastic area.^[3,10–12]^ Periosteal chondroma is found on the surface of long bones and can cause cortical erosion. It may also be confused with extraosseous osteochondroma on imaging. Tumoral calcific deposits typically appear on imaging as well-defined lobulated calcified masses that may be stratified on horizontal projection. The masses are easily distinguished by the presence of trabecular structures within them.^[3,5,11,12]^

The diagnosis of extraosseous osteochondroma is difficult based on the patient’s clinical presentation and imaging examination, and a definitive diagnosis is largely dependent on the pathological histology. Clinicians should fully consider the relevant circumstances and focus on differentiation when encountering similar cases. After completing ancillary examinations, the relationship between the mass and the surrounding tissues and blood flow should be determined before surgical treatment to avoid causing considerable trauma. As there have been no reports of malignant transformation and metastasis, most patients are expected to have an ideal prognosis after surgical local excision. In a follow-up report by Chung and Enzinger, recurrence was identified as a potential problem, and surgical excision remains the first choice for 18% of recurrent patients.^[1,5]^

## Author contributions

Shaobo Zhu collected the data, imaging, and operation reports and wrote the initial draft of the manuscript and subsequent revisions; Junhao Zeng was involved in editing and overseeing the text; Zhi Zhang was the chief surgeon of the patient; Cunmin Rong was the senior author and was responsible for oversight of the report and editing the manuscript.

**Investigation:** Junhao Zeng.

**Resources:** Zhi Zhang.

**Writing – original draft:** Shaobo Zhu.

**Writing – review & editing:** Cunmin Rong.
